# [^18^F]GE-180-PET and Post Mortem Marker Characteristics of Long-Term High-Fat-Diet-Induced Chronic Neuroinflammation in Mice

**DOI:** 10.3390/biom13050769

**Published:** 2023-04-28

**Authors:** Luisa Müller, Nicole Power Guerra, Anna Schildt, Tobias Lindner, Jan Stenzel, Newshan Behrangi, Carina Bergner, Teresa Alberts, Daniel Bühler, Jens Kurth, Bernd Joachim Krause, Deborah Janowitz, Stefan Teipel, Brigitte Vollmar, Angela Kuhla

**Affiliations:** 1 Rudolf-Zenker-Institute for Experimental Surgery, Rostock University Medical Centre, 18057 Rostock, Germany; luisa.mueller2@uni-rostock.de (L.M.);; 2 Department of Psychosomatic Medicine and Psychotherapy, Rostock University Medical Centre, 18147 Rostock, Germany; 3 Centre for Transdisciplinary Neurosciences Rostock (CTNR), Rostock University Medical Centre, 18147 Rostock, Germany; 4 Institute of Anatomy, Rostock University Medical Centre, 18057 Rostock, Germany; 5 Smell & Taste Clinic, Department of Otorhinolaryngology, Faculty of Medicine Carl Gustav Carus, Technische Universität Dresden, 01034 Dresden, Germany; 6 Core Facility Multimodal Small Animal Imaging, Rostock University Medical Centre, 18057 Rostock, Germany; 7 Institute of Anatomy and Cell Biology, Medical University of Bonn, 53115 Bonn, Germany; 8 Department of Clinic and Polyclinic for Nuclear Medicine, Rostock University Medical Centre, 18057 Rostock, Germany; 9 Department of Psychiatry, University of Greifswald, 17475 Greifswald, Germany; 10 Deutsches Zentrum für Neurodegenerative Erkrankungen (DZNE) Rostock/Greifswald, 18147 Rostock, Germany

**Keywords:** neuroinflammation, diet-induced obesity, high-fat diet, [^18^F]FDG PET/CT, [^18^F]GE-180 PET/CT, TSPO

## Abstract

Obesity is characterized by immoderate fat accumulation leading to an elevated risk of neurodegenerative disorders, along with a host of metabolic disturbances. Chronic neuroinflammation is a main factor linking obesity and the propensity for neurodegenerative disorders. To determine the cerebrometabolic effects of diet-induced obesity (DIO) in female mice fed a long-term (24 weeks) high-fat diet (HFD, 60% fat) compared to a group on a control diet (CD, 20% fat), we used in vivo PET imaging with the radiotracer [^18^F]FDG as a marker for brain glucose metabolism. In addition, we determined the effects of DIO on cerebral neuroinflammation using translocator protein 18 kDa (TSPO)-sensitive PET imaging with [^18^F]GE-180. Finally, we performed complementary post mortem histological and biochemical analyses of TSPO and further microglial (Iba1, TMEM119) and astroglial (GFAP) markers as well as cerebral expression analyses of cytokines (e.g., Interleukin (IL)-1β). We showed the development of a peripheral DIO phenotype, characterized by increased body weight, visceral fat, free triglycerides and leptin in plasma, as well as increased fasted blood glucose levels. Furthermore, we found obesity-associated hypermetabolic changes in brain glucose metabolism in the HFD group. Our main findings with respect to neuroinflammation were that neither [^18^F]GE-180 PET nor histological analyses of brain samples seem fit to detect the predicted cerebral inflammation response, despite clear evidence of perturbed brain metabolism along with elevated IL-1β expression. These results could be interpreted as a metabolically activated state in brain-resident immune cells due to a long-term HFD.

## 1. Introduction

Obesity is mainly characterized by the excessive accumulation of adipose tissue [1]. Its prevalence is steadily increasing and reaching pandemic levels, severely burdening patients and health care systems [1,2]. The main contributor inducing a detrimental increase in body weight is a permanent oversupply of food rich in calories and mostly also in fat, forming an imbalance between energy uptake and expenditure [1]. Obesity is associated with a state of chronic systemic low-grade inflammation (LGI) [3,4] affecting adipose tissue, the liver [5,6] and even the central nervous system (CNS) [7]. In this context, a decrease in cognitive abilities has been observed in patients with long-term obesity [8,9]. Additionally, obese patients have an increased risk of developing dementia [10,11,12].

The main converging mechanism of brain alterations in obesity and neurodegenerative diseases is neuroinflammation [13,14]. In obesity, neuroinflammation is predominantly found in the hypothalamus, leading to dysregulations in the hypothalamus–pituitary axis, thus promoting further disruptions in body mass and food intake regulation [15]. In addition, the literature also suggests neuroinflammatory processes in many other regions of the CNS, for example in the cortex, hippocampus and cerebellum, to be associated with neuronal loss and cognitive changes [16].

Obesity-induced neuroinflammation is thought to result from a persisting peripheral LGI characterized by changes in mediators with pro-inflammatory potential, for example leptin, saturated fatty acids and cytokines. These can affect brain myeloid cells with microglia as the most prominent CNS-resident immune cells [17] as well as astroglia by different pathways [16,18]. Various degrees of microglial and astroglial activation are also commonly observed in different neurodegenerative disorders [19,20]. The pro-inflammatory activation of microglia typically leads to a shift from a homeostatic phenotype to a pro-inflammatory state, characterized by increased expression of pro-inflammatory cytokines, such as interleukin (IL)-1β and tumor necrosis factor α (TNFα), as well as pro-inflammatory chemokines and reactive oxygen species [21]. Furthermore, the pro-inflammatory activation of microglia leads to characteristic morphological changes in which the microglia retract their fine processes, and both the cell bodies and processes become hypertrophic [22].

Besides post mortem histological analyses, neuroinflammation can be assessed in vivo using positron emission tomography (PET). The translocator protein 18 kDa (TSPO), formerly known as the peripheral benzodiazepine receptor, is a prominent biomarker for PET imaging studies [23,24]. For example, (4S)-N,N-Diethyl-9-[2-[^18^F]fluoroethyl]-5-methoxy-2,3,4,9-tetrahydro-1H-carbazole-4-carboxamide (flutriciclamide, [^18^F]GE-180 [25]) is a radiotracer with high TSPO affinity [26,27,28]. TSPO is mainly expressed in glial cells in the brain as a transmembrane protein in the outer mitochondrial membrane. While its biological functions remain largely unclear [29], several studies have shown an upregulation of TSPO expression in activated microglia, and TSPO radiotracers have been used successfully for PET imaging of neuroinflammation in various animal models as reviewed by van Camp et al. [30], including in obese mice [31].

Despite the extensive focus of research on the interconnection between obesity and neurodegeneration that is potentially mediated via neuroinflammatory processes, this is still an open field for research. In particular, publications on the effect of long-term high-caloric malnutrition on neuroinflammation are scarce. Therefore, we used a diet-induced obesity (DIO) mouse model to study the effects of a long-term high-fat diet (HFD) on brain glucose consumption and cerebral neuroinflammation as well as peripheral and cerebral markers of obesity and inflammation. We used a combination of in vivo PET analyses with 2-[^18^F]fluoro-2-deoxy-D-glucose ([^18^F]FDG) and [^18^F]GE-180 and ex vivo histological and biochemical evaluations of brain tissue. Our study aimed to test whether the application of [^18^F]GE-180 PET could detect potential chronic neuroinflammation induced by long-term HFD in the presented DIO model in vivo. Secondly, we evaluated if post mortem histological and biochemical analyses resemble cellular or humoral immune reactions induced by long-term HFD in line with the in vivo imaging results.

## 2. Materials and Methods

### 2.1. Animal Model

For the experiments, female C57BL/6J mice were purchased from Charles River (Sulzfeld, Germany) at the age of 4 weeks. Mice were kept in standard cages with 4 animals per cage in a temperature-controlled room (21 ± 3 °C) with a 12/12 h day/night cycle containing a twilight period of 30 min. After one week of acclimatization, the diet was changed to the corresponding diet. One group received a HFD (D12492; Research Diets, New Brunswick, NJ, USA), hereinafter referred to as the HFD group (16 mice) and the other group received the manufacturers’ recommended control diet (CD, D12450J; Research Diets, New Brunswick, NJ, USA), hereinafter referred to as the CD group (16 mice) over a long-term period of 24 weeks. During the experiments, mice had ad libitum access to water.

All animal experimental work was carried out with permission of the local Animal Research Committee (Landesamt für Landwirtschaft, Lebensmittelsicherheit und Fischerei (LALLF)) of the state Mecklenburg-Western Pomerania (LALLF M-V/TSD/7221.3-2-001/18) and all animals received human care according to the EU Directive 2010/63/EU.

### 2.2. PET/CT Imaging and Image Analysis

Imaging was performed after 24 weeks (=long-term) of the respective diet. For imaging procedures, radiotracers were injected into the tail vein in anesthetized mice (1.5–2.5% isoflurane Baxter, Unterschleißheim, Germany) with oxygen supplement (Air Liquide, Hamburg, Germany). Research conditions during PET imaging were kept identical among animals to ensure accuracy and reliability. These included anesthesia time (<90 min), anesthesia depth (constant breathing frequency, absence of movement) and body temperature (38 °C).

PET imaging using [^18^F]FDG was performed according to a previously published protocol [32,33]. Briefly, mice were not fasted prior PET imaging. They received 15.38 ± 0.62 MBq of [^18^F]FDG intravenously (i.v.). 30 min after injection of [^18^F]FDG, static PET imaging was performed for 30 min in the head-prone position using a small-animal PET/CT scanner (Inveon PET/CT Siemens, Knoxville, TN, USA). PET data were normalized, corrected (for attenuation, decay, scatter, randoms and deadtime) and reconstructed using 2D-ordered subset expectation maximization algorithm (2D-OSEM, 4 iterations, 16 subsets).

To investigate neuroinflammation, the radiotracer [^18^F]GE-180 was used. Briefly, mice received 15.80 ± 1.45 MBq [^18^F]GE-180 i.v. PET imaging was performed for a total of 60 min and started simultaneously with the i.v. injection of [^18^F]GE-180. All 60 min of data were averaged for analysis, and the same corrections and reconstruction algorithm as for [^18^F]FDG imaging were applied.

PET image analysis was performed with PMOD v.4.0 (PMOD Technologies, Zurich, Switzerland). Detailed procedure of PET data analysis is described by Rühlmann et al. [33]. In short, PET images were co-registered to Mirrione MRI atlas [34] using CT images and anatomical T1-weighted MRI images. The volume of interest (VOI) template of Mirrione was used to extract values in kBq/mL for the following brain regions: cortex, hippocampus and hypothalamus. Additionally, all VOIs of the template were united to obtain a whole-brain VOI. Initially, the % injected dose per mL (%ID/mL) was calculated for standardization.

**Figure 1:**
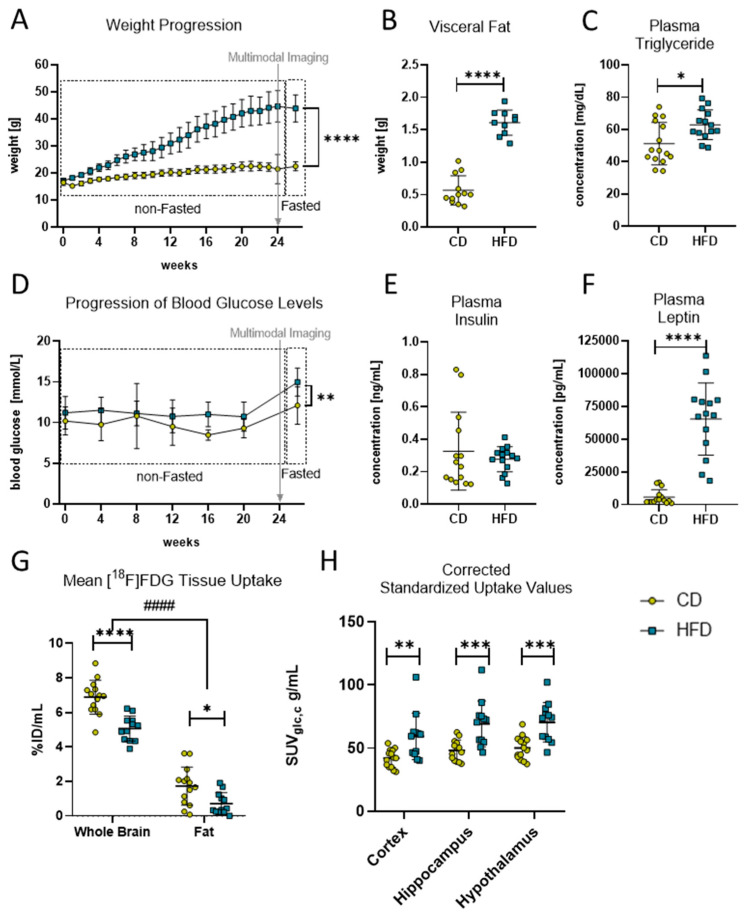
Diet-induced obese phenotype in mice. (**A**) Weekly weight progression (in g) of mice with control diet (CD) and high-fat diet (HFD). Both groups started with n = 16 mice and ended with CD (n = 15) and HFD (n = 14) mice. Last data point was measured after a fasting period of 6–12 h. Time point of multimodal imaging is indicated by the grey arrow. Data presented as group mean ± standard deviation. Statistical analysis by mixed-effects model analysis followed by post hoc tests with Sidak’s correction for multiple comparisons, **** *p <* 0.0001. (**B**) Comparison of visceral fat deposition (in g) after 24 weeks in HFD and CD group. Statistical analysis by unpaired *t*-test, **** *p <* 0.0001. (**C**) Comparison of fasted plasma triglyceride concentration (in mg/dL) after 24 weeks in HFD and CD group. Statistical analysis by unpaired *t*-test, * *p* < 0.05. (**D**) Progression of monthly measured blood glucose concentration (in mmol/L) of mice with HFD and CD. Both groups started with n = 16 mice and ended with CD (n = 15) and HFD (n = 14) mice. Last data point measured after a fasting period of 6–12 h. Time point of multimodal imaging is indicated by the grey arrow. Data presented as group mean ± standard deviation. Statistical analysis by mixed-effects model followed by post hoc tests with Sidak’s correction for multiple comparisons, ** *p <* 0.01. (**E**) Comparison of fasted plasma insulin concentration (in mg/mL) after 24 weeks in the HFD and CD group. Statistical analysis by Mann–Whitney test, *p =* 0.5409. (**F**) Comparison of fasted plasma leptin concentration (in pg/mL) after 24 weeks in HFD and CD group. Statistical analysis by Mann–Whitney test, **** *p <* 0.0001. (**G**) Comparison of tracer uptake (%ID/mL) into brain and fat tissue in volumes of interest in mice receiving CD (n = 14) or HFD (n = 12). Statistical analysis by two-way ANOVA followed by post hoc tests with Sidak’s correction for multiple comparisons, #### *p <* 0.0001 brain vs. fat, * *p <* 0.01, **** *p <* 0.0001 CD vs. HFD). (**H**) Standardized uptake values corrected for blood glucose concentration and metabolic weight (SUV_glc,c_) in g/mL obtained from [^18^F]FDG PET imaging. Depicted are cortex, hippocampus and hypothalamus as brain regions of interest of CD (n = 14) or HFD (n = 12). Statistical analysis by repeated measures ANOVA followed by post hoc tests with Sidak’s correction for multiple comparisons, ** *p <* 0.01, *** *p <* 0.001.

**Figure 2:**
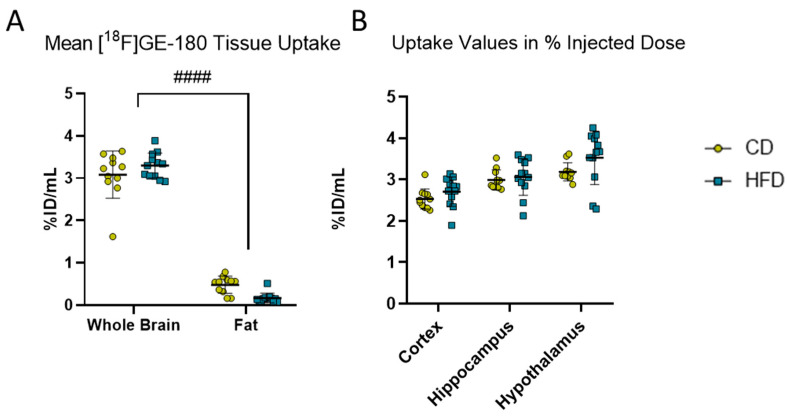
[^18^F]GE-180 PET imaging. (**A**) Comparison of mean uptake (%ID/mL) into brain and fat tissues in volumes of interest in mice receiving control diet (CD, yellow, n = 12) or high-fat diet (HFD, blue, n = 12). Statistical analysis by two-way ANOVA followed by post hoc tests with Sidak’s correction for multiple comparisons, #### *p <* 0.0001 brain vs. fat. (**B**) %ID/mL obtained from [^18^F]GE-180 PET imaging in cortex, hippocampus and hypothalamus in CD (n = 12) or HFD (n = 12). Statistical analysis by repeated measures ANOVA followed by post hoc tests with Sidak’s correction for multiple comparisons.

To address the large difference in weight between CD and HFD mice (see Section 3, Figure 1A), we evaluated the metabolic activity of each radiotracer in the visceral fat tissue and the whole brain (see Figure 1G and Figure 2A). For this, a VOI was placed manually in the visceral fat tissue. For [^18^F]FDG, the visceral fat was metabolically active to some degree (Figure 1G); therefore, we did not want to overestimate the effect of weight. Thus, we calculated the standardized uptake value using metabolic weight (SUV_c_) according to Kleiber et al. [35]. For this, the final blood glucose measurement 24 h post PET imaging was used. Additionally, SUV_c_ was corrected using blood glucose concentration (SUV_glc,c_). The following equation was used to calculate SUV_glc,c_ for [^18^F]FDG:

SUVglc,c=radioactivity concentration[kBqmL]injected dose [MBq]·body weightg·34·blood glucose[mmolL]

As for [^18^F]GE-180, the visceral fat did not show relevant [^18^F]GE-180 uptake (Figure 2A), no correction for body weight was applied and %ID/mL was used for further analysis.

### 2.3. Behavioural Test: Morris Water Maze

After 24 weeks of the respective diet, the Morris water maze (MWM) test was performed according to our own previously published work [32,36] as a measure for the mice’s spatial reference memory. After four days of acclimatization, on the fifth day (test day), time spent in the north (N) zone, frequency of platform crossing, latency to first platform crossing and velocity were monitored in real time over 180 s. For this, a video camera (15E objective, Computar, CBC Europe, Düsseldorf, Germany with camera CCA1300-60gm, Basler, Ahrensburg, Germany) was used and subsequent digital analysis was applied using Ethovision XT II.5 (Noldus Information Technology, Wageningen, The Netherlands).

### 2.4. Weight Control, Blood Sampling, Euthanasia and Tissue Preparation

Body weight was measured weekly (Kern PCB, Lübeck, Germany) and monthly retrobulbary blood sampling for glucose measurement was performed under anesthesia (5 vol.% isoflurane (Baxter, Unterschleißheim, Germany), 0.8 L/min O_2_ and 1.25 L/min N_2_O (both from Air Liquide, Hamburg, Germany)).

After PET imaging, all animals were allowed to fully recover from anesthesia in their home cages for at least 24 h. During the last six to twelve hours, mice were fasted for insulin, glucose, leptin and triglyceride measurements in plasma, followed by final body weight measurement, blood sampling and tissue collection. For this procedure, mice were deeply anesthetized and blood was taken via retrobulbary needles to exsanguinate the mice. Blood samples were kept in EDTA tubes (Microvette^®^ 500, Sarsted, Nümbrecht, Germany) at 4 °C until plasma preparation the same day.

Then, mice were transcardially perfused with 20–25 mL 0.9% NaCl (Braun, Melsungen, Germany) with an estimated flow rate of 2.28–2.83 mL/min. After perfusion, visceral fat and brains were dissected, weighted and naively snap-frozen for molecular analyses or paraffin-fixed for histological analyses.

### 2.5. Blood and Plasma Analyses

Directly after blood collection, blood glucose concentration in naïve blood samples was assessed with a glucose meter (Contour^®^XT, Bayer, Leverkusen, Germany). For plasma preparation, blood samples were centrifuged at 1200 rpm and 6 °C for 10 min (Centrifuge 5424, Eppendorf, Leipzig, Germany). Afterwards, the supernatant was collected and stored at −80 °C. From stored plasma samples, measurement of plasma triglycerides was performed using Triglyceride Colorimetric Assay Kit (Nr. 10010303, Cayman Chemical Company, Hamburg, Germany). Additionally, the stored plasma samples were used to perform insulin (Ultra Sensitive Mouse Insulin ELISA, Crystal Chem, Zaandam, The Netherlands) and leptin ELISAs (Mouse/Rat Leptin Quantikine ELISA, R&D Systems, Abingdon, UK) according to the manufacturer’s instructions.

### 2.6. Histology and Immunohistochemistry

Paraffin-embedded specimens (HFD: n = 5, CD: n = 5) were sagittally cut in 4 µm thin sections. To assess TSPO, microgliosis and astrogliosis immunohistochemical reactions were performed with primary antibodies directed against TSPO (polyclonal rabbit anti-TSPO, ab109497, Abcam, Rozenburg, The Netherlands), ionized calcium binding adapter molecule 1 (Iba1; polyclonal rabbit anti-Iba1, 019-19741, FUJIFILM Wako Pure Chemical Corporation, Neuss, Germany), transmembrane protein 119 (TMEM119; monoclonal rabbit anti-TMEM119, Abcam, Cambridge, UK) or glial fibrillary acidic protein (GFAP; monoclonal rabbit anti-GFAP, Abcam, Cambridge, UK). After deparaffination and antigen retrieval in the microwave with citrate buffer (Iba1, GFAP) or Tris-EDTA buffer (TMEM119, TSPO), blocking of endogenous peroxidases was performed with 3 % H_2_O_2_ solution. Then, slides were exposed to primary antibodies with overnight incubation at 4 °C. After incubation with secondary antibodies for 1 h at room temperature (TSPO, Iba1, GFAP: biotinylated anti-rabbit; Vector Laboratories, Biozol Di-agnostica Vertrieb GmbH, Eching, Germany; TMEM119: biotinylated anti-rabbit, EnVision, Agilent Technologies, Santa Clara, CA, USA), the avidin/biotin-based amplification kit (TSPO, Iba1, GFAP: Vectastain Elite, Biozol Diagnostica Vertrieb GmbH, Eching, Germany; TMEM119: EnVision Kit, Agilent Technologies, Santa Clara, CA, USA) was applied for one hour at room temperature. Visualization was performed with 3,3′-diaminobenzidine tetrahydro-chloride as chromogen for TSPO, Iba1 and GFAP, whereas TMEM119 antibody reactions were visualized with EnVision Kit. In the end, slides were counterstained with hematoxylin, dehydrated, mounted in DePeX (Serva Electrophoresis GmbH, Heidelberg, Germany) and coverslipped.

Recordings of immunohistochemical reactions as well as the corresponding negative controls (Supplementary Materials Figure S1) were performed with Leica DM6 B microscope equipped with a DMC6200 camera (Leica Microsystems CMS GmbH, Wetzlar, Germany). Quantitative analysis of stained cells was conducted using ImageJ (v 1.53q, Wayne Rasband, National Institutes of Health, Bethesda, MD, USA) with cell counter plugin. In addition to the cell numbers per mm^2^, the ramification index according to Zhan et al. [22] was calculated for Iba1-stained tissue as ratio of maximum projection area and maximum cell area, both measured manually.

### 2.7. Quantitative Real-Time PCR

RNA was isolated with RNeasy Mini (Qiagen, Hilden, Germany) Kit from 100 mg naïvely snap-frozen brain tissue stored at −80 °C. In addition to the manufacturer’s instructions, samples were incubated at room temperature for 5 min with 1 mL Qiazol (Qiagen, Hilden, Germany) and for 3 min with 200 µL chloroform (Sigma Aldrich, Taufkirchen, Germany) before 10 min centrifugation at 12,000× *g* at 4 °C (Centrifuge 5804, Eppendorf, Leipzig, Germany).

After isolation, RNA integrity was verified by agarose gel electrophoresis. RNA concentration was assessed by absorption measurement with NanoDrop (Thermo Fisher Scientific, Waltham, MA, USA). A total of 1 µg of the isolated RNA was transcribed into cDNA with added SuperScript™ (Invitrogen, Thermo Fisher Scientific, Waltham, MA, USA) and deoxyribonucleoside triphosphates (Thermo Fisher Scientific, Waltham, MA, USA). For further analysis, cDNA was diluted in a ratio of 1:2.

Analyses of *il-1β*, *il-6* and *tnfα* as well as *gfap* were performed via quantitative real-time PCR in a BioRad iQ5 Multicolor Real-Time PCR Detection System (Conquer Scientific, San Diego, CA, USA) with iQ™ SYBR^®^ Green Supermix (Bio-Rad, Hercules, CA, USA). Measurement results were corrected against the housekeeping gene 40S ribosomal protein S18 (*rps18*), and relative quantification was carried out by usage of the 2^−∆∆CT^ method. Primer sequences are shown in Table 1.

Furthermore, quantitative real-time PCR of *tspo* and *iba1* expression was performed using TaqMan™ Universal Master Mix II with UNG (Thermo Fisher Scientific GmbH, Dreieich, Germany) with compatible probes for *tspo* (Assay ID: Mm00437828_m1), *iba1* (Assay ID: Mm00479862_g1) and *gapdh* as housekeeping gene (Assay ID: Mm99999915_g1), according to manufacturer’s instructions (all from Thermo Fisher Scientific GmbH, Dreieich, Germany).

### 2.8. Statistical Analysis

Statistical analysis was performed using GraphPad Prism 8.0.1 (GraphPad Software Inc., San Diego, CA, USA). According to Power Guerra et al., prior tests for normal distribution were performed using Shapiro–Wilk test [37]. If necessary, outliers identified with the built-in ROUT method of GraphPad Prism with a maximum desired false discovery rate of 1% (Q = 1%) were removed from the data set. In this case, corrected animal numbers are indicated in the corresponding figure legends. For comparisons between CD and HFD groups, *t*-test (normally distributed data) or Mann–Whitney test (not normally distributed data) was performed. For evaluation of longitudinal or paired measurements, data were analyzed using either a repeated measure ANOVA or a linear stacked mixed-effects model (which in contrast to ANOVA is able to handle missing values) with diet and experimental time as fixed effects and individual mice as random effect. According to the GraphPad Prism preinstalled packages, the mixed-effects model uses a compound symmetry covariance matrix and is fit using restricted maximum likelihood (REML). Post hoc tests were performed to correct for multiple comparisons using Sidak’s method. In general, data are presented as mean ± standard deviation. Differences were deemed statistically significant at *p <* 0.05. For further details, please see figure legends.

## 3. Results

### 3.1. Long-Term High-Fat Diet Induces an Obese Phenotype with Brain Glucose Hypermetabolism

To confirm the effects of the HFD and to verify the DIO in the mice, the progression in the mice’s body weight was monitored continuously during the experimental time. The linear mixed-effects model analysis revealed a statistically significant effect of experimental time (F_(2.30, 66.98)_ = 313.0, *p <* 0.0001), diet (F_(1.00, 30.00)_ = 196.4, *p <* 0.0001) and their interaction (F_(25.00, 726.00)_ = 120.1, *p <* 0.0001) with statistically significant differences after 24 weeks of the respective diets (Figure 1A, CD: 22.5 ± 1.7 g vs. HFD: 43.9 ± 5.0 g, *p <* 0.0001). Additionally, after 24 weeks post mortem, quantifications of the dissected fat revealed a statistically significant higher mass of visceral fat (Figure 1B, CD: 0.6 ± 0.2 g vs. HFD: 1.6 ± 0.2 g, *p <* 0.0001) and plasma triglyceride concentrations (Figure 1C, CD: 51.2 ± 12.1 mg/dL vs. HFD: 62.9 ± 9.2 mg/dL, *p =* 0.013) in the HFD group compared to the CD group, confirming a DIO phenotype in this group.

Moreover, DIO led to observable derailments in metabolic pathways in the HFD group (Figure 1D–F). The linear mixed-effects model analysis revealed a statistically significant effect of diet (F_(1.00, 30.00)_ = 20.84, *p <* 0.0001) but not of experimental time or an interaction effect of both in the non-fasted blood glucose levels of the mice, with a higher average concentration in the HFD group throughout the whole experimental time compared to the CD group (Figure 1D, CD: 9.7 ± 0.8 mmol/L vs. HFD: 11.1 ± 0.3 mmol/L, *p =* 0.0026). Additionally, statistically significant elevated fasting blood glucose levels (Figure 1D, CD: 12.1 ± 2.3 mmol/L vs. HFD: 15.0 ± 1.2 mmol/L, *p =* 0.0049) but not plasma insulin levels (Figure 1E) were measured in the HFD group compared to the CD group after 24 weeks of the diets. Furthermore, the post mortem plasma analyses revealed that the HFD group had statistically significantly elevated plasma concentrations of leptin (Figure 1F, CD: 5714 ± 5596 pg/mL vs. HFD: 65,247 ± 27,496 pg/mL, *p <* 0.0001).

To determine the effects of the DIO phenotype on CNS metabolism in vivo, we performed an [^18^F]FDG-PET imaging analysis. The analysis of tracer uptake (%ID/mL) in brain tissue and visceral fat for [^18^F]FDG revealed a statistically significant effect of tissue (F_(1, 24)_ = 403.1, *p <* 0.0001) and diet (F_(1, 24)_ = 29.46, *p <* 0.0001) but no interaction effect, and post hoc tests showed a statistically significant difference between the CD and HFD in the whole-brain (*p <* 0.0001) and fat VOIs (*p =* 0.0119). Because of the measured differences between the CD and HFD in fat tissue (see methods, Figure 1G), we present SUVs corrected for the metabolic weight according to Kleiber [35] and corrected for blood glucose concentration SUV_glc,c_ when comparing glucose metabolism in the brain VOIs (Figure 1H). In doing so, after 24 weeks, we found a statistically significant effect in SUV_glc,c_ between the two diets (F_(1, 24)_ = 14.5, *p =* 0.0009) and significant differences in the cortex (CD: 42.0 ± 7.1 g/mL vs. HFD: 59.3 ± 18.4 g/mL, *p =* 0.0042), hippocampus (CD: 48.3 ± 8.1 g/mL vs. HFD: 69.1 ± 18.1 g/mL, *p =* 0.0004) and hypothalamus (CD: 50.1 ± 9.1 g/mL vs. HFD: 70.5 ± 15.6 g/mL, *p =* 0.0006).

### 3.2. Long-Term High-Fat Diet Causes Only a Change in Pro-Inflammatory Cytokine Profile

To observe the effects of the DIO phenotype on inflammation in the CNS in vivo, we performed [^18^F]GE-180 PET imaging. Comparing the activity in whole brain versus fat, the diet did not show a statistically significant effect (F_(1, 21)_ = 0.1887, *p =* 0.6685) in the mixed-effects model. However, statistically significant effects of tissue (F_(1, 21)_ = 1199.0, *p <* 0.0001) and tissue with diet interaction (F_(1, 21)_ = 10.240, *p =* 0.0043) were found, but the post hoc test did not show a statistically significant difference between the CD and HFD in the whole brain (*p =* 0.2351) or fat (*p =* 0.0572). Therefore, we refrained from further normalization steps and presented the results as %ID/mL. We analyzed the VOIs for the cortex, hippocampus and hypothalamus. Although the values of %ID tended to be higher in the HFD group (3.3 ± 0.3 %ID/mL) compared to the CD (3.1 ± 0.6 %ID/mL) group, the repeated measures ANOVA revealed a tissue-dependent (F_(1.219, 25.61)_ = 80.38, *p <* 0.0001) effect but no effect of the corresponding diet between the CD and the HFD (F_(1, 21)_ = 1.691, *p =* 0.2075).

**Figure 3:**
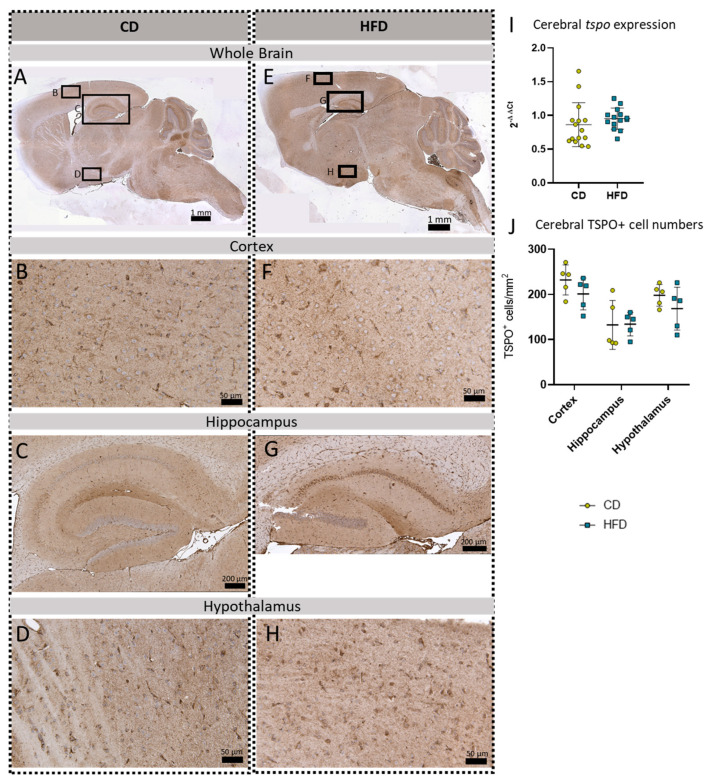
Immunohistochemical TSPO reactions in sagittal brain sections of mice after long-term (24 weeks) control diet (CD, n = 5) or high-fat diet (HFD, n = 5) within designated regions of interest (Figure **B**–**D**,**F**–**H**) defined by black rectangles in overview images (Figure **A**,**E**). (**A**) Overview of a representative sagittal TSPO-reacted brain slice of the CD group with indicated regions for detailed images; scale bar: 1 mm. (**B**) Detailed image of TSPO-reacted cortex of the CD group; scale bar: 50 µm. (**C**) Detailed image of the TSPO-reacted hippocampus of the CD group; scale bar: 200 µm. (**D**) Detailed image of the TSPO-reacted hypothalamus of the CD group; scale bar: 50 µm. (**E**) Overview of a representative sagittal TSPO-reacted brain slice of the HFD group with indicated regions for detailed images; scale bar: 1 mm. (**F**) Detailed image of the TSPO-reacted cortex of the HFD group; scale bar: 50 µm. (**G**) Detailed image of the TSPO-reacted hippocampus of the HFD group; scale bar: 200 µm. (**H**) Detailed image of the TSPO-stained hypothalamus of the HFD group; scale bar: 50 µm. (**I**) 2^−ΔΔCT^ values representing relative mRNA expression of *tspo* in the brains of CD (n = 15) vs. HFD (n = 14) group. Data presented as group mean ± standard deviation. Statistical analysis by Mann–Whitney test, *p =* 0.267. (**J**) Mean cell numbers of TSPO^+^ cells/mm^2^ in the cortex, hippocampus and hypothalamus of CD vs. HFD groups. Data presented as group mean ± standard deviation. Statistical analysis by repeated measures ANOVA with post hoc tests without corrections for multiple comparisons; cortex: *p =* 0.1905, hippocampus: *p =* 0.9546, hypothalamus: *p =* 0.2638.

**Figure 4:**
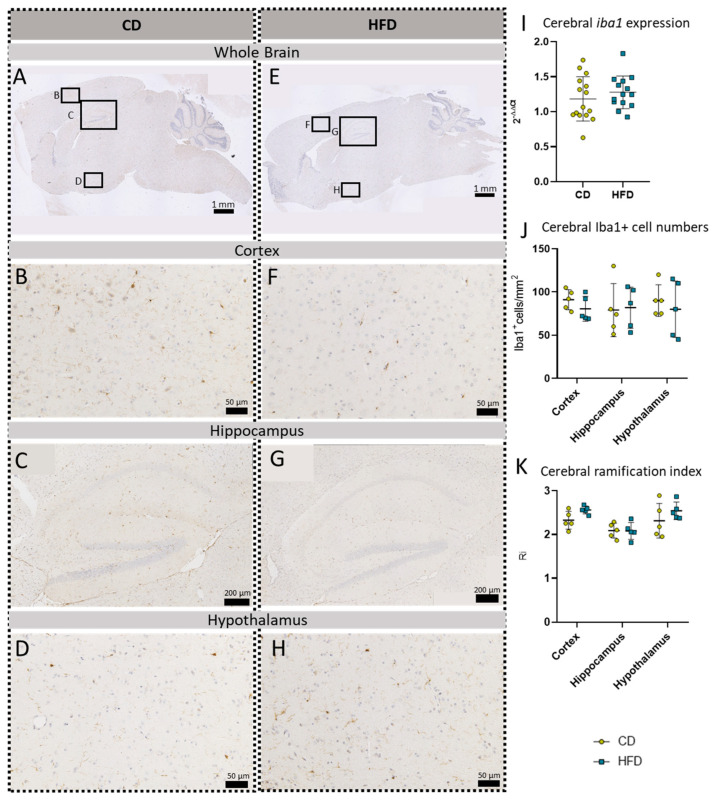
Immunohistochemical Iba1 reactions in sagittal brain sections of mice after long-term (24 weeks) control diet (CD, n = 5) or high-fat diet (HFD, n = 5) within designated regions of interest (Figure **B**–**D**,**F**–**H**) defined by black rectangles in overview images (Figure **A**,**E**). (**A**) Overview of a representative sagittal Iba1-reacted brain slice of CD group with indicated regions for detailed images; scale bar: 1 mm. (**B**) Detailed image of the Iba1-reacted cortex of the CD group; scale bar: 50 µm. (**C**) Detailed image of the Iba1-reacted hippocampus of the CD group; scale bar: 200 µm. (**D**) Detailed image of the Iba1-reacted hypothalamus of the CD group; scale bar: 50 µm. (**E**) Overview of a representative sagittal Iba1-reacted brain slice of the HFD group with indicated regions for detailed images; scale bar: 1 mm. (**F**) Detailed image of the Iba1-reacted cortex of the HFD group; scale bar: 50 µm. (**G**) Detailed image of the Iba1-reacted hippocampus of the HFD group; scale bar: 200 µm. (**H**) Detailed image of the Iba1-reacted hypothalamus of the HFD group; scale bar: 50 µm. (**I**) 2^−ΔΔCT^ values representing relative mRNA expression of *iba1* in the brains of CD (n = 15) vs. HFD (n = 14) groups. Data presented as group mean ± standard deviation. Statistical analysis by unpaired *t*-test, *p =* 0.9162. (**J**) Cerebral cell numbers of Iba1^+^ cells/mm^2^ in the cortex, hippocampus and hypothalamus of CD vs. HFD groups. Data presented as group mean ± standard deviation. Statistical analysis by repeated measures ANOVA, with post hoc tests, without corrections for multiple comparisons; cortex: *p =* 0.5699, hippocampus: *p =* 0.9981, hypothalamus: *p =* 0.9210. (**K**) Cerebral mean ramification index of Iba1^+^ cells in the cortex, hippocampus and hypothalamus of CD vs. HFD groups. Data presented as group mean ± standard deviation. Statistical analysis by repeated measures ANOVA with post hoc tests, without corrections for multiple comparisons; cortex: *p =* 0.0611, hippocampus: *p =* 0.9575, hypothalamus: *p =* 0.2951.

**Figure 5:**
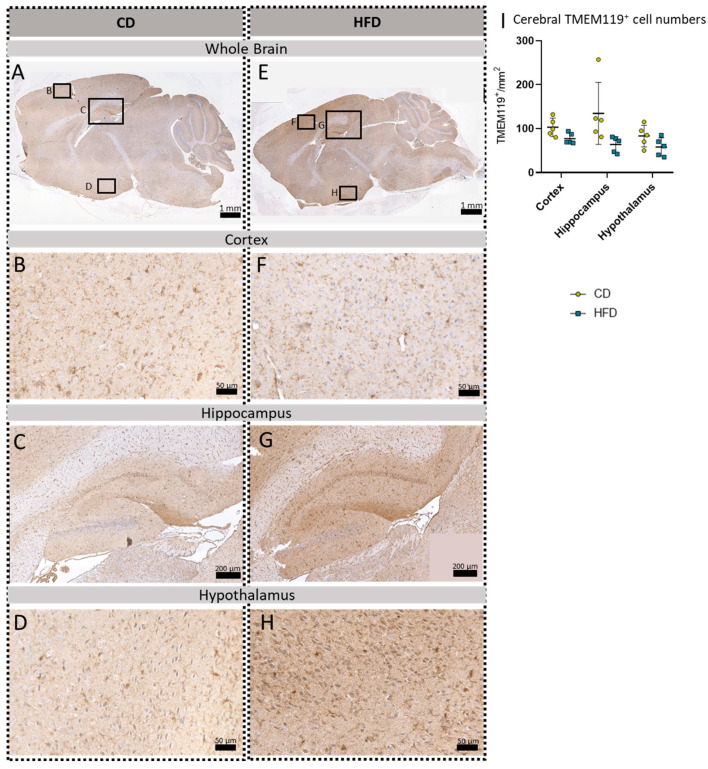
Immunohistochemical TMEM119 reactions in sagittal brain sections of mice after long-term (24 weeks) control diet (CD, n = 5) or high-fat diet (HFD, n = 5) within designated regions of interest (Figure **B**–**D**,**F**–**H**) defined by black rectangles in overview images (Figure **A**,**E**). (**A**) Overview of a representative sagittal TMEM119-reacted brain slice of the CD group with indicated regions for detailed images; scale bar: 1 mm. (**B**) Detailed image of the TMEM119-reacted cortex of the CD group; scale bar: 50 µm. (**C**) Detailed image of the TMEM119-reacted hippocampus of the CD group; scale bar: 200 µm. (**D**) Detailed image of the TMEM119-reacted hypothalamus of the CD group; scale bar: 50 µm. (**E**) Overview of a representative sagittal TMEM119-reacted brain slice of the HFD group with indicated regions for detailed images; scale bar: 1 mm. (**F**) Detailed image of the TMEM119-reacted cortex of the HFD group; scale bar: 50 µm. (**G**) Detailed image of the TMEM119-reacted hippocampus of the HFD group; scale bar: 200 µm. (**H**) Detailed image of the TMEM119-reacted hypothalamus of the HFD group; scale bar: 50 µm. (**I**) Cerebral mean cell numbers of TMEM119^+^ cells/mm^2^ in the cortex, hippocampus and hypothalamus of CD vs. HFD groups. Data presented as group mean ± standard deviation. Statistical analysis by repeated measures ANOVA with post hoc tests, without corrections for multiple comparisons; cortex: *p =* 0.0511, hippocampus: *p =* 0.0878, hypothalamus: *p =* 0.1223.

To further evaluate a pro-inflammatory cellular immune response in the brain, we used post mortem analyses of histological samples with immunohistochemical reactions to three commonly used markers for microglia: TSPO (Figure 3A–H), Iba1 (Figure 4A–H) and TMEM119 (Figure 5A–H). Furthermore, for *tspo* (Figure 3I) and *iba1* (Figure 4I), an mRNA expression analysis was performed to complement these immunohistochemical reactions. In all three reactions of the cortex, hippocampus and hypothalamus, the total number of microglia was counted as TSPO^+^cells/mm^2^ (Figure 3J), Iba1^+^cells/mm^2^ (Figure 4J) and TMEM^+^cells/mm^2^ (Figure 5I), respectively. In the Iba1-reacted tissue, the morphological activation of microglia was further assessed as the ramification index, which is the ratio between the maximum projection area and the maximum cell area (Figure 4K). Neither of the described analyses showed any statistically significant difference between the HFD and CD groups in the TSPO, Iba1 or TMEM119 reactions.

**Figure 6:**
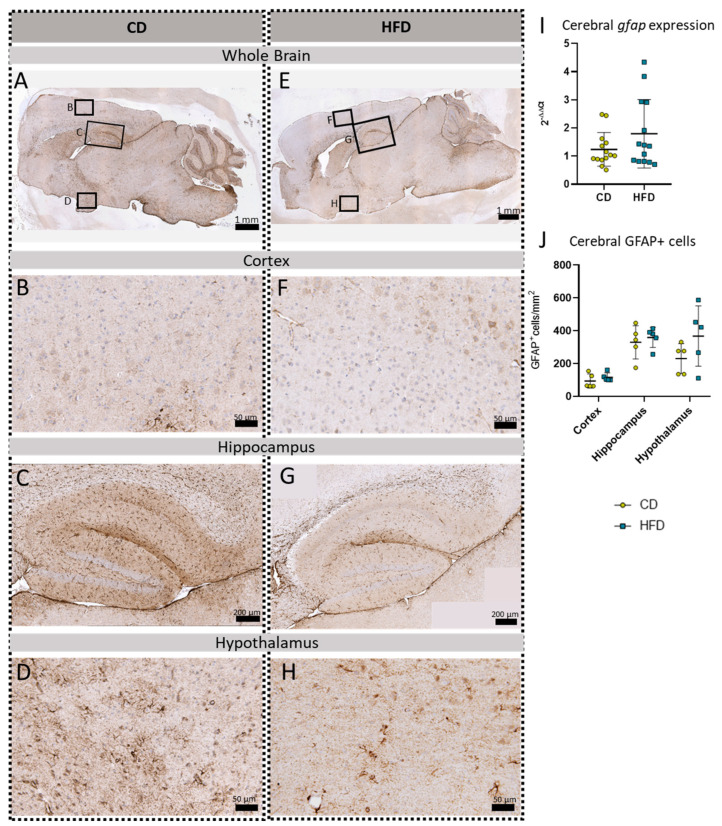
Immunohistochemical GFAP reactions in sagittal brain sections of mice after long-term (24 weeks) control diet (CD, n = 5) or high-fat diet (HFD, n = 5) within designated regions of interest (Figure **B**–**D**,**F**–**H**) defined by black rectangles in overview images (Figure **A**,**E**). (**A**) Overview of a representative sagittal GFAP-reacted brain slice of the CD group with indicated regions for detailed images; scale bar: 1 mm. (**B**) Detailed image of the GFAP-reacted cortex of the CD group; scale bar: 50 µm. (**C**) Detailed image of the GFAP-reacted hippocampus of the CD group; scale bar: 200 µm. (**D**) Detailed image of the GFAP-reacted hypothalamus of the CD group; scale bar: 50 µm. (**E**) Overview of a representative sagittal GFAP-reacted brain slice of the HFD group with indicated regions for detailed images; scale bar: 1 mm. (**F**) Detailed image of the GFAP-reacted cortex of the HFD group; scale bar: 50 µm. (**G**) Detailed image of the GFAP-reacted hippocampus of the HFD group; scale bar: 200 µm. (**H**) Detailed image of the GFAP-reacted hypothalamus of the HFD group; scale bar: 50 µm. (**I**) 2^−ΔΔCT^ values representing relative mRNA expression of *gfap* in the brains of CD (n = 15) vs. HFD (n = 14) groups. Data presented as group mean ± standard deviation. Statistical analysis by Mann–Whitney test, *p =* 0.4472. (**J**) Mean cell numbers of GFAP^+^ cells/mm^2^ in the cortex, hippocampus and hypothalamus of CD vs. HFD groups. Data presented as group mean ± standard deviation. Statistical analysis by repeated measures ANOVA with post hoc tests, without corrections for multiple comparisons; cortex: *p =* 0.3384, hippocampus: *p =* 0.5936, hypothalamus: *p =* 0.1874.

In addition to the three microglia-related immunohistochemical reactions, we also performed a *gfap* mRNA expression analysis and GFAP immunohistochemical reactions to analyze the astroglial cell numbers in the brain tissue (Figure 6). No statistically significantly increased *gfap* expression (Figure 6I) or cell numbers of GFAP^+^cells/mm^2^ (Figure 6J) in either of the presented brain regions, namely the cortex (Figure 6B,F), hippocampus (Figure 6C,G), and hypothalamus (Figure 6D,H), were observed comparing the CD and HFD groups.

**Figure 7:**
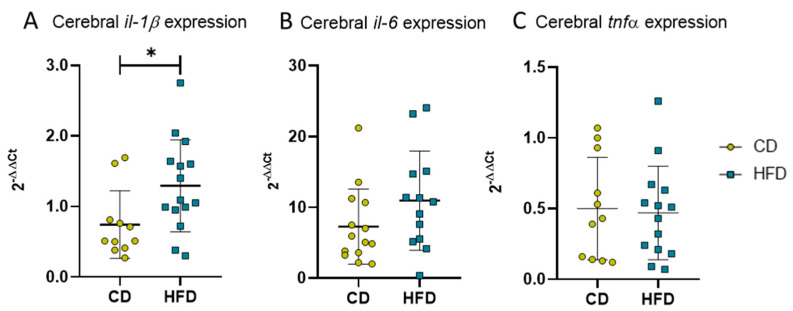
2^−ΔΔCT^ values representing relative mRNA expression of pro-inflammatory cytokines in brains of mice receiving control diet (CD) or high-fat diet (HFD) for 24 weeks. (**A**) Relative expression of *il-1β.* Statistical analysis by unpaired *t*-test, * *p <* 0.05. (**B**) Relative expression of *il-6*. Statistical analysis by Mann–Whitney test, *p* = 0.0763. (**C**) Relative expression of *tnfα*. Statistical analysis by unpaired *t*-test, *p =* 0.8261.

Besides cellular immune responses, we also analyzed cytokine expression as an indicator for the DIO-derived humoral reaction of neuroinflammation in long-term HFD mice. The PCR analysis of brain tissue revealed statistically significantly elevated levels of pro-inflammatory *il-1β* (Figure 7A, CD: 0.7 ± 0.5 vs. HFD: 1.3 ± 0.7, *p =* 0.0224) as well as a tendency of increased *il-6* (Figure 7B, CD: 7.3 ± 5.3 vs. HFD: 11.0 ± 7.0, *p =* 0.0763) but no statistically significant difference in *tnfα* expression (Figure 7C).

### 3.3. Spatial Memory Function Is Unaffected by Long-Term High-Fat Diet-Derived Changes in the Brain

**Figure 8:**
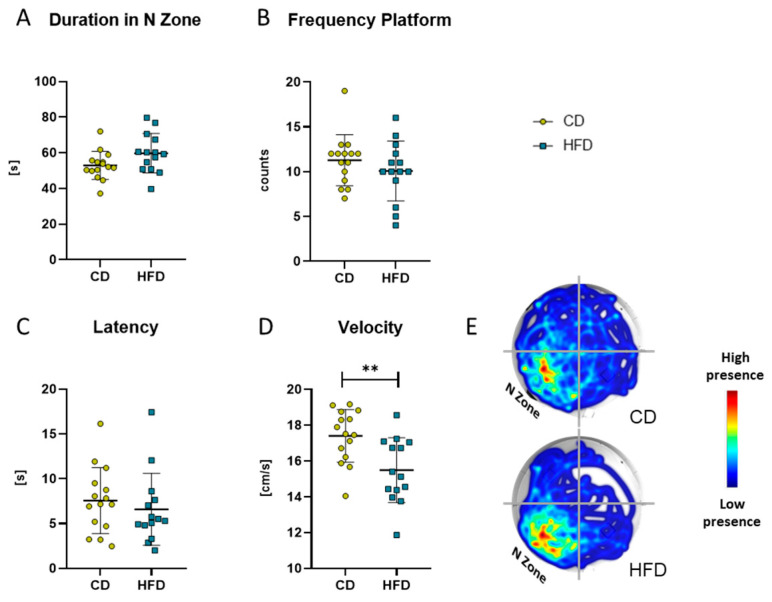
Results obtained with the Morris Water Maze test in the group receiving control diet (CD) and the group receiving the high-fat diet (HFD). (**A**) Time (in seconds (s)) spent in the N Zone, defined as quadrant containing the platform. (**B**) Frequency (counts) of platform crosses during 120 s. (**C**) Latency (s) to first platform crossing. (**D**) Velocity (cm/s) of the mice. Values are given as mean ± SD. Significance of differences between the groups was tested by unpaired Student’s *t*-test (**A**,**D**) or Mann–Whitney test (**B**,**C**), ** *p <* 0.005. (**E**) Representative heat map of the performance of mice of CD and HFD groups.

After 24 weeks of the respective diet, the MWM test was performed as a measure for spatial reference memory. There was no difference in the time the mice spent in the target N zone of the maze (Figure 8A), number of platform crossings (Figure 8B) or latency to the first crossing of the platform area (Figure 8C). Only a difference in the mean velocity was observable between the two groups (Figure 8D, CD: 17.4 ± 1.5 cm/s vs. HFD: 15.5 ± 1.8 cm/s, *p =* 0.0042).

## 4. Discussion

In the present study, we used in vivo PET imaging with the radiotracers [^18^F]FDG and [^18^F]GE-180, a TSPO ligand, to determine the effects of DIO in a long-term female HFD model on glucose metabolism and inflammatory processes in the brain, respectively. Complementarily, we performed histological and biochemical post mortem analyses of TSPO and further microglial (Iba1, TMEM119) as well as astroglial (GFAP) markers. Our main finding was a statistically significant elevation in pro-inflammatory *il-1β* mRNA expression without statistically significant changes in [^18^F]GE-180 uptake and histological correlates of induced glial proliferation and activation.

In the present study, we used female mice receiving a 60% HFD for 24 weeks (long-term). This DIO model was chosen to reflect the development of overweight and obesity due to the high-caloric diets common in Western industrialized countries. Although a high caloric proportion of Western diets also consists of sucrose, which has been shown to induce neuroinflammatory processes even in non-obese rodents [38], a high caloric intake due to an elevated fat content and its described potential to induce neuroinflammatory processes is the focus of current research approaches [39,40]. Such DIO models are generally more representative of human obesity than genetic models of obesity [41,42]. However, it should be noted that currently there are several different experimental protocols published describing the establishment of DIO using a HFD. For example, some studies describe a HFD with ~20% fat content [43], while others use the term HFD for chow containing ~50% [44,45] or ~60% fat [46,47,48]. Furthermore, the administration of HFDs to study neuroinflammation in mice has ranged from a few days [47,48,49] to several weeks [46,47,50] or a few months [31,47,50]. This underlines the need for a clear definition and a higher standardization of the different compositions and lengths of HFD administration for better comparability and reproducibility of results, even if the present study cannot achieve this either. Moreover, there is a known sexual dimorphism in the development of concomitant morbidities [51] with differences in neuroinflammatory processes between female and male DIO mice [52,53]. As there is already abundant literature regarding the effects of DIO and the interplay between metabolic syndrome and neuronal function in male animals [54], we used female C57BL/6J mice to further investigate the effect of DIO on females. Despite the possible sexual dimorphism, in line with the above-mentioned studies in male mice, we were able to show a generalized HFD-derived obese phenotype after 24 weeks of a HFD in our female model. This obese phenotype was mainly characterized by increased body weight, visceral fat, free triglycerides and leptin in plasma, as well as increased fasted blood glucose levels. Furthermore, our in vivo approach using [^18^F]FDG PET imaging showed cerebral glucose hypermetabolism in the female HFD mice compared to the CD mice. This pathological condition has already been described in male mice receiving HFD [55] and morbidly obese male and female patients [56].

In humans, obesity often has a slow onset, and the exact orchestration of the transition from an obesity-induced state of peripheral LGI to neuroinflammation is a process that is little understood and takes a long time, sometimes even decades [57]. Regarding the investigation into neuroinflammation, the literature provides plenty of insights on male mice receiving the same HFD that was used in this work with different experimental times of a few days up to a few weeks [47]. Findings from the current literature describe acute neuroinflammation after short-term HFD administration for a few days, which transiently subsides with prolonged HFD exposure; however, with chronic HFD exposure over 20 weeks (=long-term HFDs), renewed neuroinflammation becomes apparent [47]. In female mice, the available literature also describes a pro-inflammatory phenotype after 12 weeks of a HFD with ~60% fat content, albeit in a less severe manifestation compared to that of male mice [52]. In line with this, the working group of Lainez et al. suggests a protective role of ovarian hormones in this DIO model [52]. Though we cannot provide data on the estrus cycle of our mouse model, inconsistent estrogen levels might have impacted the current PET study.

Data on long-term HFDs (6 months and more), which would be a more valid representation of the long pathologic span in human obesity, and comorbidities in female mice are not yet available. This emphasizes the relevance of the present study. To observe neuroinflammation in our long-term HFD approach, we performed in vivo PET imaging with [^18^F]GE-180, a well-established marker for microglial activation in preclinical studies [26,27,28]. In our model, the results suggest a tendency for the elevated uptake of [^18^F]GE-180 in the HFD group compared to the CD group; however, these did not reach statistical significance. To further elucidate the [^18^F]GE-180 PET imaging results of the mice after 24 weeks, their brains were analyzed post mortem using molecular biology as well as histological methods. Here, we measured relative *tspo* mRNA expression in the whole brain as well as TSPO-positive cells in our three main target regions: the cortex, hippocampus and hypothalamus. None of these analyses showed any difference between the HFD and CD groups.

In general, large differences in bodyweight are an obstacle to properly analyzing [^18^F]GE-180 uptake. As we saw no relevant [^18^F]GE-180 uptake into fat tissue, we reported our results as %ID/mL. This is in line with another working group that reported good agreement between in vivo TSPO signals and immunoreactivity and suggested the use of %ID/g for quantifying TSPO in living brains [31]. Interestingly, Barron et al. found similar results to our study when using %ID/g and immunoreactivity for TSPO [31]. Barron et al. used a 12-week HFD (60% fat) model and were able to show that obesity alone did not increase TSPO inflammatory signals [31]. A possible explanation for the PET findings could be the use of the semi-quantitative measure %ID/mL for the quantification of [^18^F]GE-180. Kinetic modelling using dynamic PET data and metabolite-corrected plasma input functions are the gold standards for the quantification of radiotracers [58,59]. Nevertheless, in rodents, blood sampling can impact the physiological homeostasis [58]. The evaluation of time–activity curves in different brain regions, similar to previous work by Barron et al. [31] or Zatcepin et al. [60], can be more sensitive compared to the averaged data we used. We are aware that our averaged PET data contain perfusion and distribution phases that are not contributing to a TSPO-specific signal. However, we were not able to perform an additional dynamic reconstruction of the raw PET listmode data due to missing normalization and quantification files. Nevertheless, we obtained similar results compared to the dynamically and statically analyzed data from Barron et al. [31]. For future studies, dynamic PET data should be evaluated to increase the sensitivity of the outcome measure. A further approach for the analysis of TSPO tracer binding is the use of the cerebellum as a pseudo-reference region which has been successfully applied in humans [61]. For our model, we also applied this approach (Supplementary Material Figure S2); however, we obtained nearly the same insights that were already presented in the Section 3. A biological reason for the slightly elevated [^18^F]GE-180 uptake in the HFD group without histological signs of neuroinflammation could be a blood–brain barrier (BBB) injury induced by the HFD [62] leading to increased BBB permeability and thus to the increased uptake of [^18^F]GE-180, similar to results obtained in human multiple sclerosis patients [63,64].

To further evaluate HFD-induced neuroinflammation on a cellular level, we focused on TMEM119, a specific microglial marker [65] lacking sexual dimorphism [66], and GFAP, a marker for astroglia and reactive astrogliosis [67] that is generally more expressed in males than females [68]. Immunohistochemical reactions did not show an increase in the respective cell numbers in our HFD mice. Additionally, we observed Iba1, a marker for brain myeloid cells, with microglia as the most prominent cells [69,70]. We measured the relative *iba1* mRNA expression in the whole brain as well as Iba1-positive cells in our three main designated brain regions: the cortex, hippocampus and hypothalamus. In line with the other histological quantifications, we also found no significant differences between the CD and HFD groups. Additionally, we measured the mean ramification index of Iba1-positive microglial cells as an indicator for their activation [22] which also showed no difference between the HFD and CD groups. Nevertheless, the results in our female model are in line with the current results in other female mice, showing no increase in microglial measures after the HFD [53].

In the context of neuroinflammation, we were not able to show a cognitive decline after 24 weeks of the HFD. This is contrary to findings of de Paula et al. [62] who showed cognitive changes in male mice after a few days of a HFD. However, recent findings from a rat model using a HFD point to a sex effect on spatial memory in the MWM test, for which only male and not female rats showed a HFD-derived cognitive decline [71]. Furthermore, it is possible that our long-term HFD of 24 weeks lead to a transient state of the mitigation of neuroinflammation in female mice, which has already been described for male mice by Thaler et al. [47]. This mitigation may be mediated by a transition of brain-resident immune cells from a pro-inflammatory (in macrophages, typically characterized as M1-like) state, lately reviewed by Yunna et al. [72], to a metabolically activated phenotype, as already described for peripheral macrophages [73,74]. This metabolically activated phenotype overexpresses pro-inflammatory cytokines via the signaling pathways typically observed in M1-like macrophages, but they cannot be identified using classical cell surface markers of activation [74]. As this is in line with the presented histological analyses, we further measured cerebral cytokine expression profiles. In the CNS, we observed no increase in tnfα or il-6 expression, as these cytokines seem to have a prominent role in the peripheral mediation of HFD-derived inflammation [37,75,76]. Interestingly, our analyses revealed a statistically significant increased expression of pro-inflammatory il-1β, which is described as a key feature of the metabolically activated phenotype of macrophages [77] and is reported as a key mediator in neuroinflammation [78,79]. Therefore, IL-1β could be an indicator for a chronic metabolically activated state of neuroinflammation derived from the long-term HFD in the present experiment.

## 5. Conclusions

From these results, we conclude that in our female DIO mouse model of a long-term (24-week) HFD, cytokine signaling via IL-1β is the predominant mediator of chronic neuroinflammation as there was no observable cellular inflammation using [^18^F]GE-180 PET imaging or immunohistochemical reactions. Further experiments are needed to evaluate the exact orchestration of the phenotype changes in the brain-resident immune cells exposed to chronic long-term HFD-induced changes and possible adaption processes.

## Figures and Tables

**Table 1:** Primers used for quantitative real-time PCR.

Primer	Orientation	Sequence
*rps18*	Forward Reverse	*5′-AGGATGTGAAGGATGGGAAG-3′* *5′-TTGGATACACCCACAGTTCG-3′*
*il-1* *β*	Forward Reverse	*5′-CCCAAGCAATACCCAAAGAA-3′* *5′-TTGTGAGGTGCTGATGTACCA-3′*
*il-6*	Forward Reverse	*5′-GTTCTCTGGGAAATCGTGGA-3′* *5′-GGAAATTGGGGTAGGAAGGA-3′*
*tnfα*	Forward Reverse	*5′-ACATTCGAGGCTCCAGTGAATTCGG-3′* *5′-GGCAGGTCTACTTTGGAGTCATTGC-3′*
*gfap*	Forward Reverse	*5′-AGAAAACCGCATCACCATTC-3′* *5′-TCACATCACCACGTCCTTGT-3′*

## Data Availability

The data presented in this study are available on request from the corresponding author.

## Supplementary Materials

The following supporting information can be downloaded at: https://www.mdpi.com/article/10.3390/biom13050769/s1, Figure S1: Corresponding negative controls of the immunohistochemical reactions of (A) TSPO, (B) Iba1, (C) TMEM119 and (D) GFAP. Scale bar representing 1000 µm; Figure S2: [^18^F]GE-180 PET imaging. (A) Comparison of mean uptake (%ID/mL) cerebellum of control diet (CD, yellow, n = 12) or high-fat diet (HFD, blue, n = 12). Statistical analysis was performed by unpaired student-*t* test. (B) Ratios of cortex, hippocampus and hypothalamus to cerebellum as pseudo-reference region in CD (n = 12) or HFD (n = 12). Statistical analysis was performed by repeated measures ANOVA followed by post hoc tests with Sidak’s correction for multiple comparisons.
