# Supplementary material for: [18F]GE-180-PET and Post Mortem Marker Characteristics of Long-Term High-Fat-Diet-Induced Chronic Neuroinflammation in Mice
Source: Biomolecules. 2023 Apr 28;13(5):769. doi: 10.3390/biom13050769 (PMC10216137; doi:10.3390/biom13050769)
Supplement: Supplementary file 1 [file biomolecules-13-00769-s001.zip › biomolecules-2315020-supplementary.pdf]

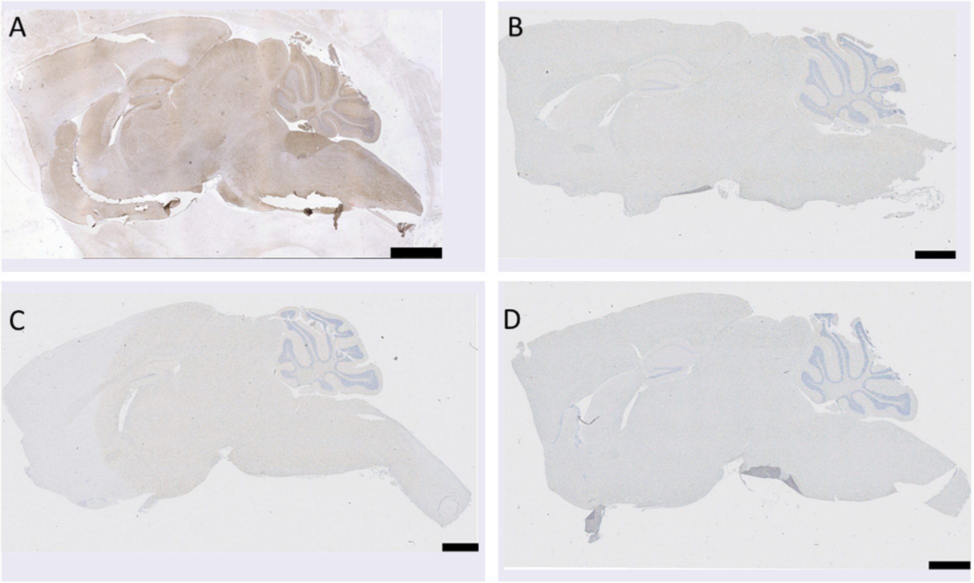

Figure S1. Corresponding negative controls of the immunohistochemical reactions of A) TSPO, B) Iba1, C) TMEM119 and D) GFAP. Scale bar representing 1000 µm.

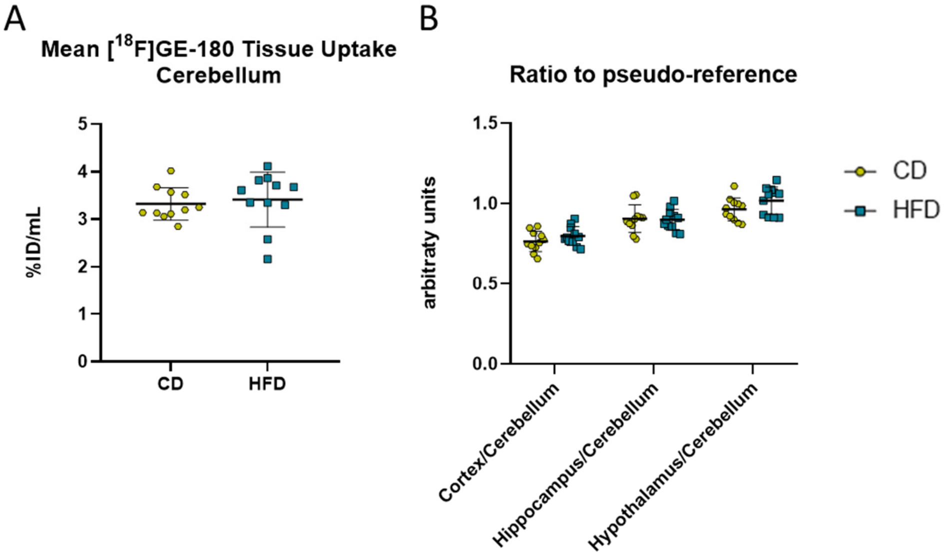

Figure S2. [^{18}F]GE-180 PET imaging. A) Comparison of mean uptake [%ID/mL] cerebellum of control diet (CD, yellow, n=12) or high-fat diet (HFD, blue, n=12). Statistical analysis was performed by unpaired student-t test. B) Ratios of cortex, hippocampus and hypothalamus to cerebellum as pseudo-reference region in CD (n=12) or HFD (n=12). Statistical analysis was performed by repeated measures ANOVA followed by post-hoc tests with Sidak´s correction for multiple comparisons.
